# Stem cell treatment for patients with autoimmune disease by systemic infusion of culture-expanded autologous adipose tissue derived mesenchymal stem cells

**DOI:** 10.1186/1479-5876-9-181

**Published:** 2011-10-21

**Authors:** Jeong Chan Ra, Sung Keun Kang, Il Seob Shin, Hyeong Geun Park, Sang Aun Joo, Jeong Geun Kim, Byeong-Cheol Kang, Yong Soon Lee, Ken Nakama, Min Piao, Bertram Sohl, Andras Kurtz

**Affiliations:** 1 Stem Cell Research Center, RNL BIO, Seoul, 153-768, Republic of Korea; 2 Otorhinolaryngology, Bethesda Samsung Hospital, Yangsan, GyeongNam Province, 626-701, Korea; 3 Graduate School of Immunology, College of Medicine, Seoul National University and Department of Experimental Animal Research, Biomedical Research Institute, Seoul National University Hospital, 101 Daehang-ro, Jongno-gu, Seoul 110-744, Republic of Korea; 4 Semyung University, 579 Sinwoul-dong, Jecheon City, Chungbuk, 390-711, Republic of Korea; 5 Kyoto Bethesda Clinic, Minami-ku, Kyoto, 601-8475, Japan; 6 Yanji Chaoyang Zaisheng Hospital, Limin Street, Chaoyangchuan Town, Yanji City, Jilin Province, China; 7 St. Mary Medical Center, OB Clinic, Long Beach, 90813, CA, USA; 8 Berlin-Brandenburg Center for Regenerative Therapies, Augustenburger Platz 1, 13353 Berlin, Germany and Seoul National University, College of Veterinary Medicine, Seoul, Korea

**Keywords:** Autologous adipose mesenchymal stem cells, autoimmune diseases, systemic stem cell infusion

## Abstract

Prolonged life expectancy, life style and environmental changes have caused a changing disease pattern in developed countries towards an increase of degenerative and autoimmune diseases. Stem cells have become a promising tool for their treatment by promoting tissue repair and protection from immune-attack associated damage. Patient-derived autologous stem cells present a safe option for this treatment since these will not induce immune rejection and thus multiple treatments are possible without any risk for allogenic sensitization, which may arise from allogenic stem cell transplantations. Here we report the outcome of treatments with culture expanded human adipose-derived mesenchymal stem cells (hAdMSCs) of 10 patients with autoimmune associated tissue damage and exhausted therapeutic options, including autoimmune hearing loss, multiple sclerosis, polymyotitis, atopic dermatitis and rheumatoid arthritis. For treatment, we developed a standardized culture-expansion protocol for hAdMSCs from minimal amounts of fat tissue, providing sufficient number of cells for repetitive injections. High expansion efficiencies were routinely achieved from autoimmune patients and from elderly donors without measurable loss in safety profile, genetic stability, vitality and differentiation potency, migration and homing characteristics. Although the conclusions that can be drawn from the compassionate use treatments in terms of therapeutic efficacy are only preliminary, the data provide convincing evidence for safety and therapeutic properties of systemically administered AdMSC in human patients with no other treatment options. The authors believe that ex-vivo-expanded autologous AdMSCs provide a promising alternative for treating autoimmune diseases. Further clinical studies are needed that take into account the results obtained from case studies as those presented here.

## Introduction

In the 21^st ^century, live expectancy has rapidly progressed as has the number of previously uncommon diseases with no treatment. Stem cell based therapies are suggested to be able to repair and regenerate tissues in diseases associated with age, changed life style and environmental exposure, such as autoimmune disease and stroke. In particular, mesenchymal stem cells (MSCs) have been applied to treat these diseases [1-3]. However, the lack of optimized culture protocols for achieving sufficient number of cells, safety issues concerning ex-vivo-expanded cells, the possible reduction in potency of stem cells derived from aged people and patients with autoimmune disease has put into question clinical applications of autologous stem cells in these patients.

In order to apply human autologous adipose tissue derived MSC (hAdMSC) in the clinical setting, we developed a standardized protocol to isolate and culture-expand AdMSC from minimal amounts of fat in vitro, achieving sufficient cell numbers for multiple therapeutic inventions [4]. Expanded AdMSCs maintained the potency for effective differentiation independently of donor age and disease status [5]. The confirmed genetic stability and in vivo safety of ex-vivo-expanded hAdMSCs in animal models and patients [4] indicate that AdMSCs from older persons are applicable for autologous therapy and are comparable to those derived from young donors [5]. Furthermore, we investigated the migration ability of hAdMSCs and their in vivo homing in animal model after systemic infusion.

MSC include a number of stem cells with an inherent ability for self-renewal and differentiation potential for mesodermal and other embryonic lineages, including adipocytes, osteocytes, chondrocytes, hepatocytes, neurons, muscle cells and epithelial cells [6-8], depending on the surrounding microenvironment. A large body of evidence demonstrated that MSC commonly have immunomodulatory and anti-inflammatory properties [9-12]. While the differentiation properties of MSC seem to dependent on microenvironmental clues in vivo, the immunomodulatory effects appear to be rather intrinsic and thus present an attractive basis for the therapy of autoimmune and inflammatory diseases by systemic infusion. Moreover, intrinsic properties of MSC demonstrated secretion of various factors, modulation of the local environment and activation of endogenous progenitor cells [13,14]. Hence, MSC therapy evoked therapeutic promises for graft-versus-host disease (GVHD), systemic lupus erythematosus (SLE), rheumatoid arthritis (RA), multiple sclerosis (MS), diabetes, myocardial infarction, thyroditis and different types of neurological disorders, among others [15-23].

Various routes of administration of MSCs, including intravenous (i.v.) [24], intraarterial [25] or intracerebral [26] were reported for stem cell application. Of these routes, i.v. is a convenient strategy to deliver cells and therapeutic effects to the injury site. Intravenously injected MSC may be transiently trapped in the lungs, sequestered in the spleen, and are predominantly eliminated by kidneys [27]. Initial accumulation of MSC in the lungs may induce secretion of secondary anti inflammatory effectors [28].The recent demonstration of in vivo homing properties of bone marrow derived MSCs and AdMSCs has further stimulated i.v. application of MSC for therapy [29]. In this review, we describe several cases of autologous AdMSCs application in autoimmune conditions, including autoimmune hearing loss, MS, polymyotitis (PM), atopic dermatitis (AD) and RA. We suggest that multiple infusions of AdMSC may establish immune homeostasis over long periods of time.

### Phenotype and differentiation potentials of MSCs

Minimal criteria have been proposed to define MSCs by the Mesenchymal and Tissue Stem Cell Committee of the International Society for Cellular Therapy. These are: 1) plastic adherence ability; 2) lack of hematopoietic markers, such as CD45, CD34, CD14, CD11b, CD79*α*, CD 19, or HLA-DR; 3) tripotential mesodermal differentiation potency into osteoblasts, chondrocytes, and adipocytes; and 4) immunomodulatory capability [30]. In addition to their mesodermal differentiation capability, MSCs were also shown to differentiate in vitro into the ectodermal lineage such as neurons, but also into the endodermal lineage such as myocytes and hepatocytes [7,31]. The conditions for differentiation of engrafted MSCs in vivo might be more complex and regulated by microenvironmental cluses of local tissues. For example, MSCs engrafted into heart could differentiate into cardiomyocytes, smooth muscle cells, and vascular endothelial cells [32-34]. In addition, through a series of signals from local tissue, engrafted MSCs can be induced to secrete diverse cytokines that posses trophic and immunomodulatory functions and subsequently contribute to tissue repair and regeneration [11].

### Sources of MSCs

MSC were first isolated as fibroblast colony-forming units (CFU-Fs) or marrow stromal cells from bone marrow (BMMSC) by Friedenstein and colleagues [35]. Their most common name is based on their property of differentiate into a variety of mesodermal tissues including bone, cartilage and fat. MSCs were found in various organs and tissues, including fat, periosteum, synovial membrane, synovial fluid, muscle, dermis, deciduous teeth, pericytes, trabecular bone, infrapatellar fat pad, articular cartilage, umbilical cord and cord blood [36,37], and placenta [38].

BMMSCs have first been applied for therapy [39,40]. However, aspirating BM from the patient is an invasive procedure that yields only low numbers of cells (about 1-10 per 1 × 10^5 ^or 0.0001-0.01% of all BM nucleated cells), requiring high expansion rates [41]. Furthermore, the therapeutic potential of BMMSCs may be diminished with increasing donor age and is associated with declining differentiation capacity and reduced vitality in vitro [42]. In any case, for autologous transplantation, expanded BMMSCs and AdMSCs have safely been applied in numerous human studies [4,39,40].

### Adipose mesenchymal stem cells

Adipose tissue is an attractive source of MSCs for autologous stem cell therapy, because adipose tissue is easily obtainable in sufficient quantities using a minimally invasive procedure [23,43]. In addition, adipose tissues contain more MSCs than BM (about 100, 000 MSCs per gram of fat) [44]. Moreover, differentiation and immunomodulatory potencies of AdMSCs are equivalent to those of BMMSCs [23].

The efficacy of AdMSCs in treating various diseases has been reported in vivo [45]. Local or systemic administration of AdMSCs was reported to have repair capacity in myocardial infarction [19] liver injury [24], hypoxia-ischemia-induced brain damage [46], allergic rhinitis [47] and muscular dystrophy [48]. Furthermore, AdMSCs are immune regulatory and potentially suitable to treat immune-related diseases including GVHD [15], MS [16], rheumatic disease [17,18] and thyroditis [20].

#### Establishment of standard culture-expansion procedure of hAdMSCs for clinical applications

Due to the small number of MSC in tissues, ex vivo expansion is required to generate the cell quantities required to achieve therapeutic results with MSCs through systemic delivery. In case of BMMSCs, however, long-term culture alters the quality of MSCs, including morphological changes, attenuated expression of specific surface markers, reduced proliferative capacity, differentiation potential [49-52], and trophic activity [53].

To produce sufficient numbers of hAdMSCs for stem cell therapy, optimized culture conditions were developed [4], which allow proliferation of hAdMSC from minimal amounts of fat since large amounts of fat are rarely obtainable from patients suffering from incurable diseases. Usage of a special cannular maximizes survival rate of stem cells in fat tissues and a 3 times higher rate of subsequent early stem cell attachment when compared to other devices. The developed cell collection, cultivation and expansion protocol requires less than 5 g fat to obtain more than 10^9 ^cells (after 3 passages). To improve proliferation and differentiation of AdMSC, we tested more than 15 commercially available culture media and eventually developed the hAdMSC culture media, named as RCME (MSC attachment media) and RKCM (MSC proliferation media) [4]. These media provide high viability, shortened doubling times and maintained morphology and improved potency.

The characteristics, stability, toxicity, and tumorigenicity of the culture-expanded hAdMSCs were determined in animals and in human studies [4]. With regard to the safety of culture-expanded stem cells in vitro, genetically stability and consistency on the morphological, immunophenotypic, and differentiation characteristics, as well as toxicity and tumorigenicity need to be verified. We demonstrated that cultured hAdMSCs showed the typical immunophenotype and differentiation capability of MSCs [4]; cells expressed MSC markers CD90, CD105, CD44 and CD29, but did not express hematopoietic or endothelial markers (CD31, CD34 and CD45) and differentiated to adipogenic, osteogenic, neurogenic, myogeneic and chondrogeneic lineages in vitro. Culture-expanded hAdMSCs were genetically stable for at least 12 passages as determined by karyotype and single nucleotide polymorphism (SNP) assays.

Cells suspended in physiological saline maintained their MSC properties, viability and potency at cold storage conditions (2 to 8°C) for at least 72 h, a critical time period for shipping stem cells into the clinic. However, we noticed that physical vibration during shipment might negatively impact cell viability. No evidence of bacterial, fungal, or mycoplasma contamination was observed in cells tested before shipping and cell viability evaluated by trypan blue exclusion was > 95% prior to cell transplantation.

#### In vivo safety of expanded hAdMSCs

To test the toxicity of hAdMSCs, different cell doses were intravenously injected into immunodeficient severe combined immunodeficiency (SCID) mice, and mice were observed for 13 weeks. Even at the highest cell dose (2.5 × 10^8 ^cells/kg body weight), mice showed no sign of discomfort. Although the safety of i.v. injection of culture expanded autologous and allogenic MSCs has been confirmed in patients [54] in numerous human clinical studies including osteogenesis imperfect [55], metachromatic leukodystrophy [56], acute myocardial infarction [57] and GVHD [58], there were some reports presenting that MSCs can induce sarcoma [59] or facilitate the growth of tumors [60]. In order to test tumorigenicity of hAdMSCs, we performed a tumorigenicity test in Balb/c-nude mice for 26 weeks. Even at the highest cell dose (2 × 10^8 ^MSCs/kg, subcutaneous injection), no evidence of tumor development was found. The safety of hAdMSCs was further investigated in a phase I human clinical trial, with no serious adverse event after i.v. administration of 4 × 10^8 ^hAdMSCs within an observation period of 12 weeks [4]. The minor adverse events found are common to spinal cord injury patients and disappeared spontaneously or were alleviated with medication. One idiopathic case of asymptomatic hyperthyroidism that did not require medical treatment remained sustained during follow-up. Based on these studies, we conclude that the systemic administration of hAdMSCs is safe and does not induce tumor development. In line with these data, Vilalta et al. [61] reported that hAdMSCs implanted in mice tended to maintain a steady state, and no detectable chromosomal abnormalities or tumors formed during the 8 months of residence in the host's tissues. Notably, the development of sarcoma in the study of Tolar *et al *was due to cytogenetically abnormal culture-expanded MSCs [59]. In addition, Izadpanah et al. [62] demonstrated that long-term cultivation of MSC beyond passage 20 may result in their transformation to malignant cells. These results indicate that it is essential to control genetic stability of culture-expanded cells.

#### Comparison of neural cell differentiation of hAdMSC derived from young and old donors

Because many diseases that are candidates for stem cells therapy are age-associated degenerative diseases, stem cells obtained from the elderly for autologous use should possess potency in order to have therapeutic effects. In terms of BMMSCs, there have been controversial results regarding the effects of aging. Using human BMMSCs from juveniles and adults seeded onto three-dimensional scaffolds, Mendes et al. [63] have demonstrated that actual bone formation decreased significantly as patient age increased. Huibregtse et al. [64] demonstrated that overall reduction in colony-forming efficiency was observed in rabbit BMMSCs derived from older animals. Bergman et al. [65] demonstrated that differences in basal proliferation rates were observed between young and old BMMSCs isolated from mice, while production of early markers of osteoblastic differentiation in vitro were equivalent. Stenderup et al. [42] have shown that human BMMSC isolated from older donors have a decreased lifespan and rate of population doubling, while both BMMSCs formed similar amounts of bone both in vitro and in vivo [51].

Adipose derived MSC seem not to undergo the same senescence pattern as BMMSC [66,67]. When hAdMSC were derived from elderly (mean 71.4 years) and young donors (mean 36.4 years), cells from both age groups showed similar proliferation, osteogenic differentiation and senescence marker patterns, while BMMSC from the same cohorts showed reduced proliferation, decreased differentiation and increased senescence [66]. In concordance with these findings are data from murine AdMSC derived from senile osteoporotic SAMP6 mice, which showed maintenance of telomere length, telomerase activity and osteogenic differentiation [67]. In order to determine the potency of hAdMSCs isolated from donors aged thirty, forty and fifty, their proliferation and differentiation potential to neural cells was investigated [5]. It was demonstrated that cell number, viability, morphology and neural differentiation potential were not different between hAdMSC of different age and passage. The results suggest that autologous adipose derived stem cells from aged people may be applied for stem cell therapy of age-dependent neural disease with the same stem cell quality and ability as stem cells derived from younger patients.

### Distribution, migration and homing potential of transplanted MSCs after intravenous injection

#### Distribution of MSCs after i.v. injection

After i.v. delivery, MSCs are generally found at low or very low frequencies in most target organs, as shown by histology, polymerase chain reaction or by immunohistochemistry [68-70]. Deak et al. [71] performed systematic kinetic assessments in non-injury models using enhanced green fluorescent protein transfected murine MSCs. They demonstrated that 24 hr after MSC application, the most frequently positive organs were lungs, liver, kidney, skin, and gut among investigated tissues. In baboons, Devine et al. [69] demonstrated that high concentration of transplant specific DNA was observed in gastrointestinal tissues. They also showed that kidney, lung, liver, thymus, and skin have relatively high amounts of DNA equivalents. Based on their studies, levels of engraftment in these tissues were estimated, ranging from 0.1 to 2.7%, with similar results with autologous and allogeneic cells [69]. After systemic administration, Lee et al. [28] found 80% of the infused MSCs in the lungs of mice 15 min after infusion, whereas after 4 days the specific signal for the presence of human MSCs decreased to 0.01%. Of importance, clinical studies with systemically delivered human MSCs did not induce significant intolerance symptoms from the pulmonary or circulatory systems, while murine MSCs displayed a somewhat different behavior. Deak et al. [72] have demonstrated in a C57BL/6 syngenic murine MSCs transfusion model, that in contrast to human MSCs, murine MSCs home to lungs and might clog in the lungs.

#### Migration and homing potential of MSCs after i.v. injection

A number of in vivo studies have shown that systemically infused MSCs could migrate to injured, inflamed tissues and exert therapeutic effects [73,74]. BMMSCs intravenously delivered to rats following myocardial infarction localize in the infarct region and improve ventricular function, while MSCs delivered to non-infarcted rats localize to the BM [75]. Localized abdomen irradiation significantly enhances MSC homing specifically to radiation-injured tissues in mice [76]. A recent study demonstrated the homing properties of i.v. administered hAdMSCs to cell-damaged areas in an allergic rhinitis animal model [47]. The relative organ distribution of fluorescence-labeled hAdMSCs was assessed by us in brain, spinal cord, spleen, thymus, kidney, liver, lung, and heart after i.v. injection in spinal cord injury rats by fluorescence microscopy and human specific *Alu *PCR. In the injured region of spinal cord, a relatively high percentage of AdMSCs (13%) was found, while most cells remained in spleen (40%) and thymus (21%) [data not shown].

Numerous studies showed the involvement of chemokines or growth factors in MSCs trafficking to the injury region. The interactions of stromal cell-derived factor-1α (SDF-1α)- and C-X-C chemokine receptor type 4 (CXCR4) mediated the trafficking of transplanted BMMSCs in a rat model of left hypoglossal nerve injury. In addition, BMMSCs were attracted by chemokines that are presented in the supernatants of primary cultures of human pancreatic islets culture *in vitro *and *in vivo *[77]. When we compared soluble factors by *in vitro *migration assay, platelet derived growth factor (PDGF)-AB and transforming growth factor-ß1 (TGF-β1) were most potent for migration activity of hAdMSCs [78]. hAdMSCs pre-stimulated with tumor necrosis factor (TNF-α) showed the highest migration activity. When analyzed by flow cytometry and reverse transcriptase-polymerase chain reaction, hAdMSC expressed C-C chemokine receptor type 1 (CCR1), CCR7, C-X-C chemokine receptor type 4 (CXCR4), CXCR5, CXCR6, EGFR (EGF receptor), FGFR1 (FGF receptor 1), TGFBR2 (TGF receptor 2), TNFRSF1A (TNF receptor 1), PDGFRA (PDGF receptor A) and PDGFRB (PDGF receptor B) at protein and mRNA levels. This study indicates that the migration of hAdMSCs is controlled by various growth factors or chemokines. Hence, modulating the homing capacity of hAdMSCs *in vivo *could stimulate its migration into injured sites after i.v. administration, and thereby improve their therapeutic potential.

### Immunomodulation and anti-inflammatory effects by MSCs

Several characteristics may play a role for the immune regulatory capability and anti-inflammatory effects of MSCs: 1) MSCs have low immunogenicity due to low expression levels of major histocompatibility complex-I (MHC-I) and no expression of MHC-II molecules and costimulatory molecules including B7-1 (CD80), B7-2 (CD86), or CD40 [79], (2) MSCs secrete soluble factors such as interleukin (IL)-6 and macrophage-colony stimulating factor [80] and suppress the activation and proliferation of T and B lymphocytes, and interfere with differentiation, maturation and function of dendritic cells, (3) MSC release anti-inflammatory and anti-apoptotic molecules and hence may protect damaged tissues [79,81].

Due to these properties, MSC transplantation has been used for the treatment of GVHD, and several autoimmune diseases, including autoimmune thyroditis [20], RA [17,18] and MS [16] and implicated for allogeneic stem cell transplantation. Systemic infusion of AdMSCs controlled lethal GVHD in mice transplanted with haploidentical hematopoietic stem cell grafts when the MSCs were injected early after transplantation [15] although ongoing clinical studies with allogeneic BMMSC were not successful. Therapeutic efficacy of BMMSCs was reported in the animal model of MS [16]. In this experimental autoimmune encephalomyelitis (EAE) model, i.v. infusion of MSCs decreased clinical symptoms when MSCs were injected before or at the onset of the disease. In an experimental collagen-induced arthritis (CIA) study, a single intraperitoneal injection of BMMSCs prevented the occurrence of severe arthritis, and was associated with a decrease in serum levels of pro-inflammatory cytokines [18]. Human AdMSCs have been demonstrated to ameliorate experimental autoimmune thyroiditis via down-regulation of Th1 cytokines [20]. Systemic infusion of hAdMSCs prevented lymphocyte infiltration to thyroid glands, decreased the production of pro-inflammatory cytokines and improved Th1/Th2 balance [20]. MSCs suppressed T-cell proliferation and cytokine production in response to alloantigen and nonspecific antigen, and prolong skin graft survival in vivo [82]. In addition, MSCs inhibit function of B cells [83], natural killer cells [84] and dendritic cells [85]. The immunomodulatory function of MSC was mediated both by soluble factors [86], and by direct cell to cell interactions [87].

Whether MSC derived from patients with autoimmune diseases will have therapeutic functions after autologous transplantation in a clinical situation is controversial and has not been addressed clinically [88]. Papadaki et al. [89] showed that while BMMSCs isolated from RA patients were found to be impaired in their ability to support hematopoiesis, BMMSCs isolated from MS patients displayed normal ability [89,90]. Other data demonstrated that BMMSCs derived from patients with RA, MS, autoimmune SLE, systemic sclerosis (SSc) and Sjogren's syndrome retained their immunomodulatory capabilities in vitro [91,92].

### Clinical application of MSCs in autoimmune diseases

Given their confirmed in vivo safety and the rationale that MSCs possess immunomodulatory and anti-inflammatory properties, compassionate-use treatments for autoimmune diseases were initiated in patients after other treatment options were exhausted. All patients provided informed consent to the treatment. Here, we describe treatment of AdMSCs in autoimmune hearing loss (AIED), MS, PM, AD and RA. Details on the patients disease and treatment histories, disease status and treatments are provided in Table 1 and Additional File 1; Case Reports, Table S1 and Figure S1. Additional clinical scores for AD before and after treatment are shown in Table 2. Patient analysis was based mostly on clinical parameters. In some cases, immunological and blood status parameters were also measured (cases 3, 4, 5, 8, 9, 10); all cases showed decrease in inflammatory responses and eosinophil counts.

**Table 1:** Summary of hAdMSC treatments of 10 patients with different autoimmune-associated diseases.

Case	Age/Sex	Injections and cell numbers	Total cell number received	Clinical status at presentation	Clinical status after treatment	Observation time (months)
***Autoimmune inner ear disease (AIED)*** *AIED *[93,94]*is a progressive, bilateral yet asymmetric, sensorineural hearing loss. Patients have higher frequencies of interferon (IFN)-c-producing T cells and higher serum antibody titres compared with healthy controls and patients with noise- and/or age-related hearing loss *[95]. *The mainstay treatment for AIED are anti-inflammatory drugs, particularly corticosteroids *[96,97]. *However, some patients are refractory to steroid treatment. Thus, alternative treatment is needed for these patients. Efficacy of hAdMSCs on experimental autoimmune hearing loss (EAHL) was shown in mice *[98]. *Mice were immunized with β-tubulin to develop EAHL and treated with i.v. injection of hAdMSCs (once a week for 6 consecutive weeks) resulting in improved hearing, hair cell stabilization, reduced proliferation of antigen-specific Th1/Th17 cells and induced anti-inflammatory cytokine IL-10 in splenocytes, induction of antigen-specific CD4(+) CD25(+) Foxp3(+) regulatory T-cells with the capacity to suppress autoantigen-specific cytotoxic T-cell responses*.						

1	19/F	3x each 2 × 10^8 ^(i.v.)	6 × 10^8^	Severe progressing hearing loss for 3 years (no in left ear, severe in right ear)	Normal hearing in right ear, moderate hearing in left ear	11

***Multiple Sclerosis (MS)*** *MS is a multifocal inflammatory disease of the central nervous system, which mainly affects young women between ages twenty and forty years and causes paralysis of the limbs, sensation, visual and sphincter problems. The disease is clinically evident with relapses of neurological disability due to damage of myelin occurs (plaques of sclerosis). The disease enters a progressive phase due to damage of the axons and irreversible neurodegeneration. Existing immunotherapies downregulate the autoimmune anti-myelin reactivity and reduced the rate of relapses (e.g. INF-β, glatiramer acetate and mitoxantrone) but progression of disability and myelin regeneration is not possible *[99,100]. *In the chronic EAE animal model *[101], *BMMSCs and AdMSCs were shown to restore neuronal activity and produce new neurons *[102,103]. *We demonstrated previously that hAdMSCs ameliorates the symptoms in EAE in a dose- and time-dependent manner, and these effects can be mediated in part by the production of anti-inflammatory cytokines *[104].						

2	46/F	5x each 1 × 10^8 ^(i.v.) 3x each 1 × 10^7 ^(intrathecal)	1.03 × 10^9^	EDSS* 8	EDSS 7	4

***Polymyositis*** *PM is a type of chronic inflammatory myopathy with unknown etiology associated with invasion of white blood cells in muscle tissue. PM is related to dermatomyositis and inclusion body myositis. Clinical signs include pain with proximal muscle weakness and loss of muscle mass, particularly in the shoulder and pelvic girdle. Despite the uncertainty in the exact cause of PM, autoimmune, viral, infectious or genetic factors have been suggested. The estimated annual incidence rate is around 5-10 cases/1, 000, 000 in the United States; it increases with age, with the highest rates seen in the 35-44 and 55-64 years. Women are two times more likely to suffer from PM than men. Corticosteroids and immunosuppressant agents are the mainstay of treatment, with a significant percentage of non-responders and clinical relapses *[105]. *Hematopoietic stem cell transplantation is performed in patients with refractory PM with satisfactory clinical efficacy *[106], *but the condition regimen for the procedure has many side effects. Allogeneic MSCs from bone marrow and umbilical cord were transplanted in 10 patients with drug-resistant PM *[107]. *Although none of the patients stopped immunosuppressive therapy for more than 1-year's follow-up and there was no cure, MSCs treatment may prove to be a useful adjunctive treatment in patients whose disease is poorly controlled with immunosuppressive agents*.						

3	35/F	4x each 5 × 10^8 ^(i.v.)	2 × 10^9^	inability to walk slope and to stand up by herself	Able to step up stairs (< 10 cm) and walk gentle slope holding handrail	3

***Atopic Dermatitis*** *AD is a common, chronic and refractory skin disease manifesting as eczema and pruritus with repeated exacerbations and regressions and unknown pathogenesis *[108]. *The incidence of AD in adults has increased worldwide over the past decade *[109]. *Current management aims to relieve frequency of dermal inflammation and prevent its flare-up using topical corticosteroids and tacrolimus *[109,110]. *Although these treatments might control the symptoms, relapse is frequent and extensive and prolonged use of corticosteroid carries risk of side-effects, including skin atrophy and there are many AD patients with corticosteroid phobia *[111]. *Despite the immunomodulating effect of MSC, there is no previous record of stem cell treatment of AD*.						

4	27/F	3x each 2 × 10^8 ^(i.v.)	6 × 10^8^	SCORAD index 93.1	SCORAD* index 61.1	5.5

5	33/M	3x each 2 × 10^8 ^(i.v.)	6 × 10^8^	SCORAD index 57.0	SCORAD index 35.5	4.5

6	27/F	5x each 2 × 10^8 ^(i.v.)	1 × 10^9^	SCORAD index 33.4	SCORAD index 16.4	3.5

7	26/F	3x each 2 × 10^8 ^(i.v.)	6 × 10^8^	SCORAD index 39.1	SCORAD index 13.3	2

***Rheumatoid Arthritis*** *RA is a T-cell-mediated systemic autoimmune disease caused by loss of immunologic self tolerance and characterized by synovium inflammation and articular destruction. MSCs were reported to reduce inflammatory and T cell responses and induce antigen specific regulatory T cells in vitro in rheumatoid arthritis *[112]. *Systemic infusion of hAdMSCs significantly reduced the incidence and severity of experimental arthritis induced by CIA in vivo *[113], *which was mediated by down-regulating Th1-driven autoimmune and inflammatory responses and induction of interleukin-10 in lymph nodes and joints. Human AdMSCs also induced de novo generation of antigen-specific CD4+CD25+FoxP3+ Treg cells. The best therapeutic benefits were seen when the stem cell treatments were performed prior to onset and by systemic rather than local application. Recently, the therapeutic effects of systemic infusion human umbilical cord (UC)-MSCs were also verified in the collagen-induced arthritis model *[114]*with effects similar to those of hAdMSCs*.						

8	50/F	2x each 3 × 10^8 ^(i.v.)	6 × 10^8^	***VAS score: 10 KWOMAC score: 73	VAS score:2-3 KWOMAC score: 28	7

9	51/F	Once 2 × 10^8^ (i.v.) + 1 × 10^8^ (intrarticular) Once 3.5 × 10^8 ^(i.v.) + 1.5 × 10^8^ (intrarticular))	8 × 10^8^	Inability to stand up, crutches for walking	Ability to stand up, off steroids	3

10	67/M	4x each 2 × 10^8 ^(i.v.)	8 × 10^8^	Inability to walk	Normal walking, off steroids	13

Detailed clinical case reports are provided in the Additional File 1 Case Reports. Multiple sclerosis: *EDSS is expanded disability status scale. Atopic dermatitis: The outcome was evaluated by the area of skin lesions, **SCORAD (SCORing Atopic Dermatitis) index [115,116] and CBC count. The changes of SCORAD index of each patient before and after AdMSCs treatment are summarized in table 1. Rheumatoid arthritis: ***VAS (Visual Analogue Scale) KWOMAC (Korean Western Ontario McMaster). Further information on patient profile and treatment for AIED are summarized in Additional File 1 Figure S1.

**Table 2:** SCORing results of AD patients.

Patient	Gender	Age	Total cell dose	Injection Route	Follow-up (months)	Extent		Intensity		Pruritus/Insomnia		Total score	
						
						Pre	Post	Pre	Post	Pre	Post	Pre	Post
1	F	27y	6 × 10^8^	Intravenous	5 1/2	98	98	17	11	14	3	93.1	61.1
2	M	33y	6 × 10^8^	Intravenous	4 1/2	75	75	7	5	14	3	57	35.5
3	F	27y	1 × 10^9^	Intravenous	3 1/2	12	7	8	4	3	1	33.4	16.4
4	F	26y	6 × 10^8^	Intravenous	2	18	4	7	3	11	2	39.1	13.3

SCORing Atopic Dermatitis (SCORAD) index in atopic dermatitis patients before and after the stem cells treatment (see also Additional File 1, Case Reports).

For all treatments, 5 g of fat tissues were collected by liposuction, transferred immediately to the GMP facility and Stem Cell Research Center of RNL BIO and culture-expanded for 3 passages using the standard protocol to obtain AdMSCs [4]. The patients received between 1 and 6 i.v. infusions of 200 million AdMSCs suspended in physiological saline (each 100 million cells/100 ml) in different intervals (see Table 1 and Additional File 1; Case Reports). Two patients received additional intrathecal (MS-patient) and intrarticular (RA-patient) injections of cells (Table 1 and Additional File 1; Case Reports and Table S1).

## Conclusions

Human AdMSC can be isolated from small amounts of adipose tissue, efficiently expanded to achieve more than 10^9 ^cells after 3 to 4 passages independent on donor age and disease status. The sustained potency and genetic stability of the cells make adipose tissue a very attractive source for multipotent cells. Their immunmodulatory function, homing and migratory patterns as well as previous clinical trials suggest that these cells are efficient for treatment for several classes of autoimmune diseases and their application is safe. Here, we demonstrated considerable therapeutic effects of culture-expanded autologous AdMSCs in a variety of autoimmune diseases in the frame of an ethically justified compassionate use application for patients with exhausted therapeutic options. Multiple intravenous infusions of cells resulted in clinical benefit in all treated patients in the follow up period. No adverse events were observed. The data provide first evidence for clinical benefit in autoimmune diseases, yet further scrutiny in controlled clinical trials with a sufficient numbers of patients are needed to draw a definitive conclusion on therapeutic efficacy and long term benefit. Importantly, the data show that multiple AdMSC infusion of up to 1 × 10^9 ^cells in a period of less than one month is safe, corroborating data from preclinical and clinical trials using BMMSC and AdMSC. Furthermore, within this small sample size, no evidence of donor age-dependent efficacy, or age dependent in vitro cell expansion rate was found. The autologous stem cell application described here is based on the current state of the art and provides an outlook into treatments for patients suffering from a variety of incurable autoimmune related diseases with no remaining treatment options. While it is shown here that the technology for treatment of autoimmune using autologous AdMSC is in place and the expectations derived from preclinical studies can be confirmed, there is still a limited understanding of the modes of action. In conclusion, the systemic infusion of autologous stem cells described here offers promise for better management of a wide spectrum of autoimmune diseases, independent on patient's age.

## Competing interest Statement

Jeong Geun Kim, Byeong Chul Kang, Yong Soon Lee, Ken Nakama, Min Piao, Betram Sohl and Andras Kurtz have no competing financial or personal interests in this work. Jeong Chan Ra, Sung Keun Kang, Il Sub Shin and Hyeong Geun Park are employees and shareholders of RNL BIO Limited, which holds patents on some of the technologies in this manuscript. San Aun Joo is employee of RNL BIO and declares no competing financial interests.

## Authors' contributions

JCR: involved in the preparation and in vitro and in vivo characterization of AdMSCs and designed the protocol of stem cell treatment and managed the clinical cases along with clinicians. SAJ: designed the protocol of stem cell treatment and cared the patients. NK: cared and treated autoimmune patients. MP: cared and treated autoimmune patients. BS: cared autoimmune inner ear disease patient and evaluated the data. SKK: performed the migration and homing potential of AdMSCs and drafted and revised the manuscript. ISS: designed and performed characterization of AdMSCs including distribution, immunophenotyping and in vitro differentiation of AdMSCs. HGP: cultured AdMSCs and performed in vitro safety and stability test of AdMSCs. JGK: analyzed and evaluated the data of autoimmune inner ear disease. BKK: performed the experiments of in vivo safety of AdMSCs. YSL: designed and evaluated data of safety and distribution of AdMSCs. AK: drafted and revised the manuscript, organized and evaluated data. All authors read and approved the final manuscript

## Supplementary Material

Additional file 1 **Individual case reports, Table S1, and Figure S1**. The file contains detailed clinical case reports for each of the treated patients with autoimmune hearing loss (AIED), multiple sclerosis (MS), polymyotitis (PM), atopic dermatitis (AD) and rheumatoid arthritis (RA). Table S1 shows the manual muscle test (MMT) grading, grading scheme for manual muscle test (MMT) in patients with MS and PM. Figure S1 shows the audiograms and conduction test for patient with AIED. Audiograms are shown for left and right ears before and after AdMSC treatment. Click here for file
