# Supplementary material for: Stem cell treatment for patients with autoimmune disease by systemic infusion of culture-expanded autologous adipose tissue derived mesenchymal stem cells
Source: J Transl Med. 2011 Oct 21;9:181. doi: 10.1186/1479-5876-9-181 (PMC3222617; doi:10.1186/1479-5876-9-181)

Additional Information

Additional File 1

Case Reports

Case 1 (AIED)

Case history: In August 2007, a 17-year-old woman consulted with an otolaryngology doctor for left sensorineural hearing loss, which started approximately 2 years earlier. In November 2008, she had sudden bilateral hearing loss and was treated with Prednisone 60 mg po (per oral) QD (once a day) for 1 month and then tapered off Valtrex po x 2 weeks. In January 2009, she was diagnosed with AIED and treated with Dyazide one tab po qAM (each morning) and Lipoflavonoids. Pulsed doses of i.v. Solumedrol 100 mg were given. After referring to a rheumatologist specializing in autoimmune hearing loss, she was treated with Plaquenil 200 mg po BID (twice a day) and Azulfadine 500 mg po BID. In February 2009, a second rheumatologist was consulted, Plaquenil and Azulfadine were stopped and treatment started with Methotrexate 15 mg po and Leucovorin 5 mg weekly along with Solumedrol 1 g daily x 3 days every month. In March 2009, Methotrexate and Leucovorin was discontinued, but continued on i.v. Solumedrol 1 g daily for 3 days once a month and treated with Humira 40 mg i.m. every 2 weeks until July 17, 2009.

Treatment and outcome: The patients received 3 times i.v. injection of $2x10^8$ AdMSCs on July 27, August 3 and 10, 2009 (total $6x10^8$ cells). Hearing ability was tested by audiometry using 3-divided classification. Before stem cell treatment (July 27, 2009), pure-tone hearing threshold of the right ear was 83 dB, indicating severe hearing loss, and of the left ear was scaled out, indicating non-measurable deafness. In air conduction test, the threshold of the right ear at 125, 250, 6000 and 8000 Hz was scaled out and left ear showed non-measurable deafness. In bone conduction test, the response of the right and left ear was 30 and 45 dB at 250 and 500 Hz, respectively. About three months after the first stem cell injection (October 16, 2009), pure-tone hearing threshold of the right ear was 22 dB, indicating normal hearing and of the left ear was 62 dB, indicating moderately severe hearing loss. In air conduction test of the right ear, she had normal hearing of 20-25 dB at all frequency except 8000 Hz. At 8000 Hz, air conduction threshold of the right ear was 50 dB, indicating moderate hearing loss. In bone conduction test, the right ear showed normal hearing. The threshold of the left ear in air and bone conduction test was 45-50 dB at below 1000 Hz, indicating moderate hearing loss. At over 2000 Hz, the threshold was 80 dB, indicating severe hearing loss. Eleven months after

stem cell treatment (June 29, 2010), pure-tone hearing threshold of the right ear was 30 dB, indicating mild hearing loss and of the left ear was 17 dB, indicating normal hearing. Taken together, the stem cell treatment improved the hearing of the right ear from severe sensorineural hearing loss to mild sensorineural hearing loss (from 83 dB to 30 dB) and of hearing of the left ear from non-measurable deafness to normal hearing (scaled out to 15 dB). (Fig. 1)

Case 2 (MS)

Case history: The 46 year old female patient was diagnosed in 1992 with a relapsing-remitting MS. Symptoms temporally improved after treatment with high-dosage steroids in the incipient stage in 1992. When presented in April 2009, she suffered from tonic spasms, stiffness, astasia, severe pain in her body and fatigue. She couldn't stand by herself and was using the wheelchair, had urination and defecation disorders. Her expanded disability status scale (EDSS) score was 8.

Treatment and outcome: Between June 16 and July 11, 2009, she was treated with five i.v. infusions once weekly each $2x10^8$ AdMSC (total 10^9 cells) and three intrathecal injections of each 1×10^7 AdMSCs. Four months after cell injection, the patient had improved EDSS by 1 point, was able to stand for 2 to 3 min and walk approximately 10 meters with assistance. Manual Muscle Test (MMT) and electromyography was measured after 1 month and after 4 month post AdMSCs treatment. Grading of MMT is described in supplementary table 2. Flexion was increased from 1 (T) to 2 (P) in the hip. Sensory and motor amplitudes in median nerves of the left arms were amplified from 58.4 to 73.8 and from 66.7 to 72.7, respectively. The motor latencies in tibial and fibular nerves of left leg were shortened from 4.65 to 3.65 and from 5.75 to 3.95, respectively. The motor conduction velocity in tibial and fibular nerves of left leg was prolonged from 47.7 to 50.0 and from 42.4 to 53.9. Her subjective energy level and mood increased at 4 months after the beginning of the AdMSCs treatment. Cytokine levels (pg/ml) before (April, 2009) and after (August, 2010) autologous AdMSCs treatment was compared. The level of IL-10 (<0.38), IL-12p70 (<0.65), IL-17 (<0.04), IL-2 (<0.02) and IL-4 (<0.08) did not change and of IFN-γ was slight increased (from 0.39 to 1.66). The level of GM-CSF (from 333.25 to <0.49), IL-1β (from 687.88 to <0.46), IL-6 (from 3322.05 to <0.77) and TNF-α (from 64.25 to 5.86) decreased dramatically.

Case 3 (Polymyositis)

Case history: The 35 years old female patient suffered from PM since 1998. She was treated with Solondo (80 mg, daily). She deteriorated with loss of muscular strength, persisting muscular complaints, and inability to walk slope or stair, or to stand up by herself. She had side effects of steroids such as weight gain, osteroporesis (T-score < 3), interruption of menstruation, pollakiurea, thin skin because she took the steroid for long duration.

Treatment and outcome: Between March and April, 5 x 10^8 AdMSCs were i.v. injected four times every 2 weeks (total 2 x 10^9 cells). MMT, laboratory blood examination and quality of life (SF-36) were performed before and 3 months after AdMSCs treatment. Flexion and extension of wrist increased from 4 to 5, and extension in knee and flexion in ankle increased from 3 to 4 and 4 to 5, respectively. No significant change was detected in the blood laboratory values. Her quality of life improved sufficiently to step up stairs (< 10 cm), walk in gentle slope holding the handrail. Also, she was able to shower, put on the clothes and drive by herself. She continued to take Solondo (20 mg, daily).

Case 4 (Atopic Dermatitis)

Case histoy: The 27 years old female patient suffered from AD since early infancy. By July 2009, the AD lesions diffused widely into scalp, face, ventral and dorsal body, and four-limbs and the degree was severe. The lesions included erythema, papulation, discharge, acne, lichenification, and asteatosis. She had also severe pruritis, which disturbed daily life and even provoked sleep disorder. The SCORAD (Scoring Atopic Dermatitis) index was total 93.1 points.

Treatment and outcome: The patient received 3 times i.v. injection of 2 x 10^8 AdMSCs on Sep 26, 2009, Nov 10 and Nov 18, 2009 (total 6x10^8 cells). Five and half months after the first injection, the AD symptoms were re-evaluated. The severity of symptoms improved to intermediate or mild; pruritis decreased as far as not to disturb her sleep. The post-injection SCORAD index was total 61.1 points. Eosinophils count in CBC test decreased from 26.6 to 5.8%. The patient was able to stop corticosteroid medications.

Case 5 (Atopic Dermatitis)

Case history: The 33 years old male patient showed AD symptoms since 2004 and had a history of asthma and allergic rhinitis. By Sep 2009, widely diffused skin lesions were found on the face, ventral and dorsal body, and four-limbs. The severity of erythema and

lichenification was evaluated at 2 points, papulation, discharge, acne and asteatosis at 1 point. He had severe pruritis disturbing daily life and provoking sleep disorder. The SCORAD index was total 57.0 points.

Treatment and outcome: The patient received 3 times i.v. injection of 2 x 10^8 AdMSCs on Sep 29, Nov 10 and Nov 18, 2009 (total 6x10^8 cells). Follow-up was performed 4.5 months after the first injection. Erythema, lichenification and asteatosis improved with reduction of one point each. Pruritis was weakened with decreased frequency of unconscious scratching. The post-injection SCORAD index was total 35.5 points. Eosinophils count in CBC test decreased from 14.1 to 6.5 % after stem cells injections; Drug medication continued.

Case 6 (Atopic Dermatitis)

Case history: The 27 years old female patient suffered from AD since infancy and had skin lesions at face, scalp, ventral and dorsal body, and arms when presenting. In December 2009, the severity of erythema and population was evaluated at 2 points each, that of discharge, acne, lichenification, and asteatosis was 1 point each. Although unconscious scratching existed, she had no sleep disorder and no medical prescriptions for AD. The SCORAD index was total 33.4 points.

Treatment and outcome: The patient received 5 times i.v. injection of 2 x 10^8 of AdMSCs on Dec 20, 2009, Dec 29, 2009, Jan 5, 2010, Jan 12, 2010, and Jan 19, 2010 (approximately 1 week interval; total 1x10^9 cells). The status of AD was re-evaluated at 3.5 months after the first injection. All skin lesions were reduced and severity of erythema, papulation, lichenification, and asteatosis was lowered to each 1 point and there was no evidence of discharge and acne. Pruritis was decreased from 3 points to 1 point. The post-injection SCORAD index was total 16.4 points. Specific alterations in CBC were not found.

Case 7 (Atopic Dermatitis)

Case history: The 26 years old female patient suffered from AD for over twenty years. Prior to stem cell treatment (July 2009), skin lesions were found at the ventral and dorsal body, and four limbs. The patient had severe pruritis disturbing daily life and provoking sleep disorder. The SCORAD index was total 39.1 points and the patient was given medication (antihistamines).

Treatment and outcome: The patient received 3 times i.v. injection of 2 x 10^8 AdMSCs on Sep 26, Nov 18 and Dec 2 (total 6 x 10^8 cells). The patient was re-evaluated 2 months after first

stem cell treatment. The skin lesions were generally reduced and no more pulpulation and discharge found. Pruritis weakened with decreased frequency of unconscious scratching and sleep disorder disappeared. The post-injection SCORAD index was total 13.3 points. The patient was able to stop drug medications.

Case 8 (Rheumatoid arthritis)

Case history: The 50 years old female suffered from joint pain for twenty years. Symptoms such as joint swelling, pain and anchylosis were evoked in her early thirty's. She was diagnosed with rheumatoid arthritis a year after the disease's onset. Based on the diagnosis, the patient was treated with steroids and anti-rheumatic drugs: Somerone Tab 0.5T SID (one a day), Sazopin Tab 1T BID, Hyroquin Tab 1T BID, Procton Tab 1T BID, Folic acid Tab 1T SID, Camillan Tab 1T BID, Methotrexate 10 mg daily. The dosage of steroids was adjusted based on the patient's symptoms. Artificial joint replacement operations were also performed 3 times at the left knee, the left wrist and the right ankle, respectively between 1995 and 2004. Prior to stem cell treatment, she experienced difficulty in performing simple daily activities due to pains in joint and swelling of knuckles, wrists, elbows, shoulders and ankles. When the joint lesions were touched or used, severe pain occurred, resulting in difficulties in grasping objects. For this case, the VAS (Visual Analogue Scale) score was 10 and the KWOMAC (Korean Western Ontario McMaster) score was 73. The results of the 36-item Short-Form (SF-36) Health Survey, widely used instrument for measuring health status are as follows; PF (physical functioning) was 10, RP (role limitations due to physical health) was 0, BP (bodily pain) was 0, GH (general health) was 10, VT (vitality) was 0, SF (social functioning) was 0, RE (role limitation due to emotional problems) was 0, and MH (mental health) was 12. C-reactive protein (CRP) was 1.4 mg/dL, rheumatoid factor (RF) was 330.9 IU/mL, and anti-CCP was over 600 U/mL.

Treatment and outcome: On February 20, 2010, the patient received the first i.v. injection of 3 x 10^8 AdMSCs. Two months after the first stem treatment, her joint pains and swellings significantly disappeared. She was able to grasp with her fingers and bend them backwards. An additional 3 x 10^8 MSCs were injected on June 11, 2010. She could wear shoes with heels for the first time in two decades and attained an increase in pain-free walking distance of over 100 meters. Seven months after the first stem cell treatment, the VAS score and KWOMAC score decreased from 10 to 2-3 and from 73 to 28, respectively. The post treatment results of SF-36 showed significant improvement in quality of life; from 10 to 50 for PF, from 0 to100

for RP, from 0 to 55 for BP, from 10 to 75 for GH, from 0 to 68 for VT, from 0 to 75 for SF, from 0 to 100 for RE, and from 12 to75 for MH. RF was slightly decreased to 272.6 IU/mL (CRP: 2.5 mg/dL, anti-CCP: > 600 U/mL). The patient was able to control pains in the joints and to stop drug medications without joint swellings occurring.

Case 9 (Rheumatoid arthritis)

Case history: The 51 years old female suffered from joint pain for over 25 years. Severe joint pain, anchylosis and swellings were found at the fingers, wrists and knees. Arthrosis deformans of fingers existed at presentation. The patient was on crutches for 7 years because her mobility was limited by joint pain. She couldn't stand up by herself on a toilet seat. Leg numbness and pains caused insomnia. Steroids and analgesics have been occasionally used for a short period. Prior to stem cell treatment, elevated levels of CRP (0.7 mg/dl), RF (184 IU/ml), and anti-CCP (27 U/ml) were observed.

Treatment and outcome: From March to April, 2010, the patient received 2 times injections of AdMSCs: Intravenous injection of 2.0 x 10^8 cells (10^8 cells/100ml saline) + intrarticular (into finger, wrist, elbow, knee joints) injection of 1.0 x 10^8 cells (5 x 10^7 cells/2.5 ml physiological saline) and intravenous injection of 3.5 x 10^8 cells + intrarticular 1.5 x 10^8 cells. 8 x 10^8 AdMSCs were injected in total. Three months after the first stem cell treatment, levels of CRP and anti-CCP returned to normal (from 0.7 mg/dl to 0.01 mg/dl, from 27 U/ml to 6.7 U/ml, respectively) and RF levels decreased from 184 IU/ml to 161.8 IU/ml. After stem cell treatments, leg numbness and pain disappeared and daily activities were easier due to decreased joint pain. She is able to use toilet and get dressed by herself.

Case 10 (Rheumatoid arthritis)

Case history: A 66 years old male was experiencing acute pain in the wrist in the midst of performing surgery on Sep 5, 2009. A few days later, the pain progressed into his knees to the extreme that he could no longer walk. He was diagnosed with autoimmune arthritis with elevated levels in CRP (14.03 mg/dl) and RF (17 IU/ml). Due to the symptoms, he was unable to perform his surgical duties and was forced to cancel 70% of his surgeries. To alleviate the pain, he took cortisone, a steroidal hormone. Although cortisone helped lower his CRP and RF levels to a normal range, the drug has led to adverse side effects such as chest pain and esophagitis.

Treatment and outcome: Starting from May 2010, he received 4 times i.v. injection of 2 x 10^8

AdMSCs in intervals of one month (total $8x10^8$ cells). At 5 months after the first stem cell treatment, he felt that his conditions improved remarkably and he stopped taking his steroidal and immunosuppressant (methotrexate) drugs. He had no symptoms and was able to resume his surgical practices. Despite the discontinuance of steroidal drugs, recent blood tests in June 2011 revealed that his CRP and RF levels were successfully maintained at 0.20 mg/dl and 8 IU/ml, respectively.

Additional File 1

Supplementary Table 1. Manual muscle test (MMT) grading.

Grade	Scale	% of disability	Definition
5	Normal (N)	0	Muscle can hold test position against strong pressure
4	Good (G)	1~25	Hold position against moderate pressure
3	Fair (F)	26~50	Hold resistance against gravity but not extra pressure
2	Poor (P)	51~75	Ability to move in partial range of motion
1	Trace (T)	76~99	Feeble contraction in muscle
0	Zero (Z)	0	No muscle contraction

Additional File 1

Supplementary Figure 1. Audiograms before and after stem cell injections in the patient with AIED (case 1). The hearing ability was tested before stem cell injection, and at 2 months and 11 months after the first stem cells injection. Air and bone conduction tests were performed on both ears.

Left ear air conduction test

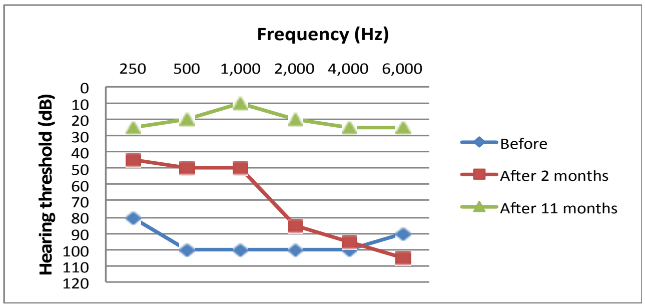

Right ear air conduction test

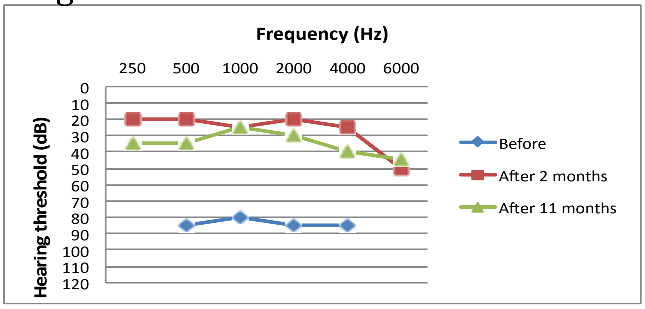

Left bone conduction test

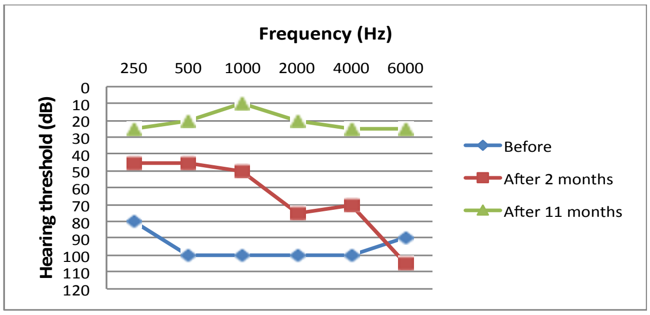

Right bone conduction test

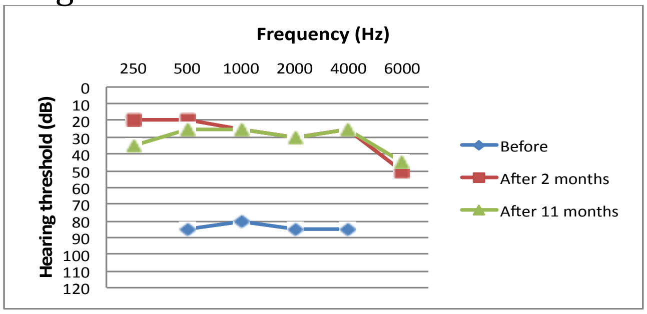

Supplement: Additional file 1 — Individual case reports, Table S1, and Figure S1. The file contains detailed clinical case reports for each of the treated patients with autoimmune hearing loss (AIED), multiple sclerosis (MS), polymyotitis (PM), atopic dermatitis (AD) and rheumatoid arthritis (RA). Table S1 shows the manual muscle test (MMT) grading, grading scheme for manual muscle test (MMT) in patients with MS and PM. Figure S1 shows the audiograms and conduction test for patient with AIED. Audiograms are shown for left and right ears before and after AdMSC treatment. [file 1479-5876-9-181-S1.PDF]
